# A tailored health surveillance program unveils a case of MALT lymphoma in an HCV-positive health-care worker

**DOI:** 10.3892/ol.2012.1028

**Published:** 2012-11-15

**Authors:** VENERANDO RAPISARDA, ANDREA MARCONI, SAVERIO CANDIDO, DARIA NICOLOSI, MARIO SALMERI, PIETRO GANGEMI, LIDIA PROIETTI, DEMETRIOS A. SPANDIDOS, MASSIMO BRACCI, CETTINA FENGA, MASSIMO LIBRA

**Affiliations:** 1 Section of Occupational Medicine, Department of Internal Medicine and Systemic Diseases, University of Catania, I-95125 Catania;; 2 Department of Biomedical Sciences, Section of Pathology and Oncology, Laboratory of Translational Oncology and Functional Genomics, University of Catania, I-95124 Catania;; 3 Pathology Unit, Vittorio Emanuele Hospital, I-95100 Catania, Italy;; 4 Department of Virology, Medical School, University of Crete, Heraklion 71003, Greece;; 5 Department of Clinical and Molecular Sciences, Occupational Medicine Section Polytechnic University of Marche, I-60020 Ancona;; 6 Section of Occupational Medicine, Department of Social and Environmental Medicine, University of Messina, I-98125 Messina, Italy

**Keywords:** hepatitis C virus, health-care worker, non-Hodgkin lymphoma, surveillance

## Abstract

Non-Hodgkin lymphoma (NHL) may occur among hepatitis C virus (HCV)-infected individuals. HCV is one of the most common blood-borne pathogens transmitted from patients to health-care workers (HCWs). The development of NHL among HCV-infected HCWs has recently been shown. To investigate this issue further a tailored health surveillance program was applied to 3,138 HCWs from four Medical Institutions. To this aim, all employees were screened for both anti-HCV antibodies and HCV-related extrahepatic manifestations. The HCV prevalence rate, similar among all the HCW subgroups, was 7.3%. The occurrence of a gastric mucosa-associated lymphoma tissue (MALT) lymphoma, diagnosed in a physician following a long history of HCV chronic infection, was observed. Molecular characterization of MALT tissue indicated that immunoglobuline gene combinations were those usually found among HCV-associated lymphomas. Furthermore, B-cell expansion exhibited t(14;18) translocation, as a genetic abnormality associated with the development of MALT lymphomas from HCV-positive patients. Overall, these findings support the hypothesis that HCV viral infection potentially affects the pathway of transformation and progression of lymphoma cells. The occurrence of B-cell NHL, among HCV-positive HCWs, is an additional reason to apply the standard precautions to reduce the risk of blood-borne pathogen transmission.

## Introduction

Health-care workers (HCWs) may be exposed to hepatitis C virus (HCV), one of many blood-borne pathogens, in their work environment (1). Previous studies (2–4) investigated the risk factors involved in HCV. HCV transmission has been shown to occur after 3–10% of percutaneous exposure to HCV (2–4). Percutaneous HCV transmission occurs following needle-stick injures or cuts from other sharp instruments. Transmission may also occur following exposure of the eyes, nose, mouth, or broken skin to HCV (5–7).

In a previous study, we showed a statistically significant decrease in the number of occupational blood exposure accidents documented in 10 years during a health surveillance program for 403 HCWs from a single institution (8). The data suggested that the guidelines including the standard precautions to reduce the risk of blood-borne pathogen transmission have been shown to have a strong efficacy among HCWs. Concomitantly, an HCV-positive HCW developed a liver mucosa-associated lymphoma tissue (MALT) lymphoma. Furthermore, molecular characterization of the tumour indicated that lymphoma was associated with the HCV chronic infection (8).

In the last two decades, studies have shown that HCV infection may contribute to the development of non-Hodgkin lymphoma (NHL) in addition to hepatocellular carcinoma, as previously demonstrated (9,10). In a systematic review of 66 studies with a focus on HCV infection and NHL development, Negri *et al*(10), reported that the majority of these studies were conducted in Italy, and their findings indicated a high prevalence of HCV infection among NHL cases, ranging from 8.9 to 37.1%. Accumulating evidence supports a model in which chronic stimulation of B-cells by antigens associated with HCV infection causes non-malignant B-cell expansion that may evolve into B-cell NHL (11).

To clarify this issue, a tailored health surveillance program was applied to 3,138 HCWs from four Medical Institutions. All HCWs were screened for anti-HCV antibodies and for HCV-related extrahepatic manifestations.

## Materials and methods

### Subjects

Subjects included 3,138 employees of four Italian Public Hospitals, i.e., Policlinico Universitario, Ferrarotto-Alessi, Santo Bambino and Ospeadali Riuniti di Torrette, Ancona. A previous series of 403 HCWs by Marconi *et al*(8) was not included in the present study. All the subjects were HCWs at high risk for exposure to blood-borne pathogens. Lifestyle risk factors for HCV infection were assessed by ascertaining whether subjects had lived with an HCV-positive partner, had been given a blood transfusion, had a history of casual sexual intercourse, had any tattoos, or had a history of intravenous drug abuse. Peripheral blood obtained from the subjects was screened for anti-HCV antibodies by an enzyme-linked immunosorbent assay (Ortho Diagnostic Systems, Raritan, NJ, USA), as previously described (12). HCWs were also examined for HCV-related malignancies. The study was approved by the University of Catania Ethics Committee. Written informed consent was obtained prior to enrolment.

### Analysis of B-cell clones

To determine B-cell clonality in the NHL sample from the HCV-positive HCW, complementary determining region-3 (CDR3) of the Ig heavy chain gene was amplified by PCR. The upstream primer was complementary to framework region-3 (FR3) of VH and the downstream primer was complementary to JH. Ig heavy chain gene DNA was amplified by PCR with upstream primers complementary to framework region-1 (FR1) of each VH gene segment family and a downstream primer complementary to CDR3 (12). PCR products were purified by gel electrophoresis, then sequenced. The most similar VH and DH germline gene segments were identified by sequence comparison to the International Immunogenetics Database with DNAplot software (http://imgt.cines.fr).

### Analysis of t(14;18)-(IgH;Bcl-2) translocation

DNA was isolated from tumour samples by standard phenol-chloroform extraction. t(14;18)-(IgH;Bcl-2) translocation, at the major break point region (MBR) and minor cluster region (mcr), was assessed by the polymerase chain reaction (PCR), as previously reported (13). AccuPrime™ SuperMix (Invitrogen, Carlsbad, CA, USA) was used to increase the specificity and sensitivity of PCR analysis. The sensitivity of our assay was 10^−5^.

PCR products were separated by electrophoresis on 2.5% agarose gel. Positive and negative control samples were included throughout all steps of the experimental procedures. Single bands obtained by amplification of the MBR and mcr from tumour biopsy specimens were purified from the gel and then sequenced on an ABI 310 Genetic Analyzer (Perkin–Elmer, Foster City, CA, USA), as previously reported (13).

### Immunophenotyping

Paraffin sections were used for immunophenotyping and lineage assignment of the NHL case. Sources and specificities of the antibodies used in this study have been reported in detail previously (14).

## Results

### Subjects

The HCWs examined included 1,352 (43%) nurses, 953 (30%) physicians and surgeons, 833 (27%) other employees (laboratory technicians, midwives and rehabilitation therapists). HCV infection was detected in 229 (7.3%) HCWs. The frequency of HCV infection according to professional categories revealed that 8.18% of physician and surgeons, 7.25% of nurses, and 6.36% of other employees were HCV-positive. The remaining 2,826 HCWs were HCV-negative (Table I). None of the HCV-positive HCWs experienced accidental blood exposure while working in the last 10 years.

The case of a 58-year-old HCV-positive male physician diagnosed in 2011 with a gastric MALT lymphoma was examined. To determine whether HCV infection is associated with the development of the B-cell lymphoproliferation, a molecular analysis of the B-cell clone was performed. Neoplastic B-cell expansion expressed VH3-7-DH6-6-JH4 Ig heavy chain genes according to the IMGT database. Furthermore, the t(14;18)-(IgH;Bcl-2) translocation, recently detected in a fraction of HCV-associated Malt lymphomas as an additional molecular marker, was analysed (14). Accordingly, a positive PCR reaction was obtained using the MBR-specific primers, indicating that the translocation involved the MBR. Nucleotide sequence analysis confirmed that Bcl-2 was joined to JH6. The breakpoint was detected at position 3,128 of the Bcl-2 gene and at position 1,504 of the JH6 gene.

### Immunophenotyping

**Figure 1.**
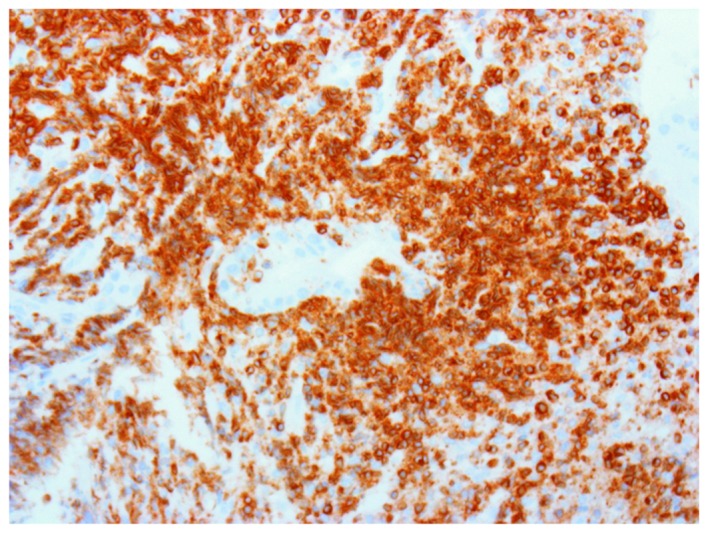
Bcl-2 immunostaining of gastric MALT lymphoma harboring t(14;18) translocation.

Tumours analyzed were CD20^+^, cyclin D1^−^, CD23^−^, CD5^−^, Bcl-6^−^, CD43^−^, and CD10^−^, supporting the diagnosis of MALT lymphoma. In particular, the null expression of CD10 and Bcl-6 excluded the follicular origin of this tumour. Moreover, the overexpression of Bcl-2 suggested that t(14;18) translocation, usually linked to HCV-associated lymphomas, may sustain survival of B-cells preventing apoptosis (Fig. 1).

## Discussion

Infection by HCV leads to the development of hepatic (9) and extra-hepatic disorders (11). In a previous study conducted in a group of 403 HCWs from a single institution a case of NHL was identified among HCV-infected employees (8). Consequently, we analysed the occurrence of B-cell lymphoma in HCV-positive HCWs from four Medical Institutions, i.e., Policlinico Universitario, Ferrarotto-Alessi, Santo Bambino and Ospeadali Riuniti di Torrette, Ancona.

The HCV prevalence rate in HCWs was 7.3%. Among the professional subgroups, the category of physician-surgeon had the highest prevalence at 8.18%, followed by that of nurse at 7.25%. The prevalence detected in the HCWs was lower than that observed in the general population suggesting that HCV infection is common in the elder population as a result of past iatrogenic transmission such as blood transfusion or surgical intervention (15). However, our results are comparable to those from other studies on the seroprevalence of HCV among HCWs, and show that the prevalence is similar among subgroups of HCWs (8,16).

Epidemiological and experimental studies have demonstrated that HCV infection contributes to the development of B-cell NHL. The prevalence of anti-HCV Abs in NHL patients was 19.7%, ranging from 8.3 to 37.1% (see 11 for a review). Based on a previous observation by Marconi *et al*(8), results of the present study conducted on 229 HCV-infected HCWs demonstrated the occurrence of B-cell lymphoma. It was a gastric MALT lymphoma, diagnosed in a physician after a long history of HCV chronic infection. Of note, the heavy chain gene combinations detected in the DNA from MALT tissue were those usually found in the HCV-associated lymphomas sustaining the role of HCV infection in the mechanism of lymphomagenesis (17). The sequence analysis of rearranged Ig genes in malignant B-cells from HCV-positive patients revealed that certain combinations of heavy and light chain genes are frequently present. These common combinations include: IGHV3-23/IGHD3-22/IGHJ4, IGHV1-69/IGHD3-22/IGHJ4 or IGHV4-59/IGHD2-15/IGHJ2 with either IGKV3-20/IGKJ1 or IGKV3-20/IGKJ2, and IGHV3-7/IGHD3-16/IGHJ3 or IGHV3-7/IGHD3-22/IGHJ3 with IGKV3-15/IGKJ1 (18–20). The overexpression of Bcl-2 detected in this MALT tissue suggests that t(14;18) translocation, linked to HCV-associated lymphomas (14), may sustain the survival of B-cells preventing apoptosis. Additionally, the immunohistochemical evaluation revealed that the null expression of CD10 and Bcl-6 excludes the follicular origin of this tumour. This finding is in agreement with previous studies as follicular lymphoma histotype is uncommon among HCV-infected patients (11,21).

Overall, these findings support the hypothesis that HCV infection affects the pathway of transformation and progression of lymphoma cells. The occurrence of B-cell NHL, among HCV-positive HCWs, is an additional reason to apply the standard precautions to reduce the risk of blood-borne pathogen transmission (22–24).

## Figures and Tables

**Table I.** Hepatitis C virus infection status according to professional categories.

Professional categories	Age (years) median (range)	HCV-positive/tested	Prevalence (%)
Nurse	53 (31–64)	98/1,352	7.25
Physician-surgeon	54 (26–69)	78/953	8.18
Other (32–62 years)	46 (32–65)	53/833	6.36
Total HCWs	51 (26–69)	229/3,138	7.26

HCWs, health-care workers.

## References

[1] Butsashvili M, Kamkamidze G, Kajaia M, Morse DL, Triner W, Dehovitz J, McNutt LA (2012). Occupational exposure to body fluids among health care workers in Georgia. Occup Med (Lon).

[2] Riddell LA, Sherrard J (2000). Blood-borne virus infection: the occupational risks. Int J STD AIDS.

[3] Torbati SS, Guss DA (1999). Emergency department management of occupational exposures to HIV-infected fluids. J Emerg Med.

[4] Beltrami EM, Williams IT, Shapiro CN, Chamberland ME (2000). Risk and management of blood-borne infections in health care workers. Clin Microbiol Rev.

[5] Klein RS, Freeman K, Taylor PE, Stevens CE (1991). Occupational risk for hepatitis C virus infection among New York City dentists. Lancet.

[6] Kiyosawa K, Sodeyama T, Tanaka E (1991). Hepatitis C in hospital employees with needlestick injuries. Ann Intern Med.

[7] Mitsui T, Iwano K, Masuko K (1992). Hepatitis C virus infection in medical personnel after needlestick accident. Hepatology.

[8] Marconi A, Candido S, Talamini R, Libra M, Nicoletti F, Spandidos DA, Stivala F, Proietti L (2010). Prevalence of hepatitis C virus infection among health-care workers: A 10-year survey. Mol Med Rep.

[9] Bartosch B, Thimme R, Blum HE, Zoulim F (2009). Hepatitis C virus-induced hepatocarcinogenesis. J Hepatol.

[10] Negri E, Little D, Boiocchi M, La Vecchia C, Franceschi S (2004). B-cell non-Hodgkin’s lymphoma and hepatitis C virus infection: a systematic review. Int J Cancer.

[11] Libra M, Polesel J, Russo AE, De Re V, Cinà D, Serraino D, Nicoletti F, Spandidos DA, Stivala F, Talamini R (2010). Extrahepatic disorders of HCV infection: A distinct entity of B-cell neoplasia?. Int J Oncol.

[12] Libra M, Gloghini A, De Re V (2005). Aggressive forms of non-Hodgkin’s lymphoma in two patients bearing coinfection of Epstein-Barr and hepatitis C viruses. Int J Oncol.

[13] Libra M, De Re V, De Vita S, Gasparotto D, Gloghini A, Rupolo M, Degan M, Marzotto A, Stivala F, Carbone A, Boiocchi M (2003). Low frequency of bcl-2 rearrangement in HCV-associated non-Hodgkin’s lymphoma tissue. Leukemia.

[14] Libra M, Gloghini A, Malaponte G (2008). Association of t(14;18) translocation with HCV infection in gastrointestinal MALT lymphomas. J Hepatol.

[15] Di Stefano R, Stroffolini T, Ferraro D, Usticano A, Valenza LM, Montalbano L, Pomara G, Craxì A (2002). Endemic hepatitis C virus infection in a Sicilian town: further evidence for iatrogenic transmission. J Med Virol.

[16] Montella M, Crispo A, Grimaldi M, Ruffolo P, Ronga D, Izzo F, Mastro AA (2005). An assessment of hepatitis C virus infection among health-care workers of the National Cancer Institute of Naples, Southern Italy. Eur J Public Health.

[17] Libra M, Gasparotto D, Gloghini A, Navolanic PM, De Re V, Carbone A (2005). Hepatitis C virus (HCV) infection and lymphoproliferative disorders. Front Biosci.

[18] De Vita S, De Re V, Sansonno D, Sorrentino D, Corte RL, Pivetta B, Gasparotto D, Racanelli V, Marzotto A, Labombarda A, Gloghini A, Ferraccioli G, Monteverde A, Carbone A, Dammacco F, Boiocchi M (2000). Gastric mucosa as an additional extrahepatic localization of hepatitis C virus: viral detection in gastric low-grade lymphoma associated with autoimmune disease and in chronic gastritis. Hepatology.

[19] Ivanovski M, Silvestri F, Pozzato G, Anand S, Mazzaro C, Burrone OR, Efremov DG (1998). Somatic hypermutation, clonal diversity, and preferential expression of the VH 51p1/VL kv325 immunoglobulin gene combination in hepatitis C virus-associated immunocytomas. Blood.

[20] Gasparotto D, De Re V, Boiocchi M (2002). Hepatitis C virus, B-cell proliferation and lymphomas. Leuk Lymphoma.

[21] Talamini R, Montella M, Crovatto M (2004). Non-Hodgkin’s lymphoma and hepatitis C virus: a case-control study from northern and southern Italy. Int J Cancer.

[22] Centers for Disease Control and Prevention (CDC) (1998). Recommendations 5 for prevention and control of hepatitis virus (HCV) infection and HCV-related chronic disease. MMWR Recomm Rep.

[23] Centers for Disease Control and Prevention (CDC) (1997). Recommendations for follow-up of health-care workers after occupational exposure to hepatitis C virus. MMWR Morb Mortal Wkly Rep.

[24] Haiduven DJ, DeMaio TM, Stevens DA (1992). A five-year study of needlestick injuries: significant reduction associated with communication, education, and convenient placement of sharps containers. Infect Control Hosp Epidemiol.

